# Willingness to accept and participate in a *Microsporidia MB*-based mosquito release strategy: a community-based rapid assessment in western Kenya

**DOI:** 10.1186/s12936-024-04941-y

**Published:** 2024-04-20

**Authors:** Tullu Bukhari, Joseph Gichuhi, Oscar Mbare, Victoria A. Ochwal, Ulrike Fillinger, Jeremy K. Herren

**Affiliations:** Human Health, International Centre of Insect Physiology and Ecology (Icipe), Nairobi, Kenya

**Keywords:** *Microsporidia MB*, Willingness to accept, Community engagement, Transmission-blocking strategy, Mosquito release

## Abstract

**Background:**

*Microsporidia MB*, an endosymbiont naturally found in *Anopheles* mosquitoes inhibits transmission of *Plasmodium* and is a promising candidate for a transmission-blocking strategy that may involve mosquito release. A rapid assessment was carried out to develop insight into sociodemographic factors, public health concerns, and malaria awareness, management, and prevention practices with the willingness to accept and participate in *Microsporidia MB*-based transmission-blocking strategy to develop an informed stakeholder engagement process.

**Methods:**

The assessment consisted of a survey conducted in two communities in western Kenya that involved administering a questionnaire consisting of structured, semi-structured, and open questions to 8108 household heads.

**Results:**

There was an overall high level of willingness to accept (81%) and participate in the implementation of the strategy (96%). Although the willingness to accept was similar in both communities, Ombeyi community was more willing to participate (OR 22, 95% CI 13–36). Women were less willing to accept (OR 0.8, 95% CI 0.7–0.9) compared to men due to fear of increased mosquito bites near homes. Household heads with incomplete primary education were more willing to accept (OR 1.6, 95% CI 01.2–2.2) compared to those educated to primary level or higher. Perceiving malaria as a moderate or low public health issue was also associated with a lower willingness to accept and participate. Experience of > 3 malaria cases in the family over the last six months and knowledge that malaria is transmitted by only mosquito bites, increased the willingness to accept but reduced the willingness to participate. Awareness of malaria control methods based on mosquitoes that cannot transmit malaria increases the willingness to participate.

**Conclusion:**

The study showed a high level of willingness to accept and participate in a *Microsporidia MB*-based strategy in the community, which is influenced by several factors such as community, disease risk perception, gender, education level, knowledge, and experience of malaria. Further research will need to focus on understanding the concerns of women, educated, and employed community members, and factors that contribute to the lower disease risk perception. This improved understanding will lead to the development of an effective communication strategy.

**Supplementary Information:**

The online version contains supplementary material available at 10.1186/s12936-024-04941-y.

## Background

Insecticide-treated bed nets (ITNs) and indoor residual spraying (IRS) contributed significantly to the reduction of malaria cases and deaths during 2005–2015 [1]. However, the overall impact of malaria control initiatives has plateaued partly because of the limitations of ITNs and IRS. This emphasizes the necessity to broaden the present malaria control toolkit to reach the goal of malaria elimination [2–5]. Transmission-blocking strategies can complement current control tools and are inherently suitable for the elimination and maintenance phases, which are characterized by a low infective mosquito population, low parasite prevalence, and focal malaria transmission [6, 7]. Currently, transmission-blocking strategies include drugs, vaccines, and refractory mosquitoes. Refractoriness can be due to genetic modification or natural and when released into the environment, these mosquitoes can suppress or replace wild malaria-susceptible mosquitoes [6, 8–10].

Community acceptance and participation are important for every vector control intervention, [11–15]. Community acceptance is a multifaceted construct that reflects the extent to which people delivering or receiving an intervention consider it appropriate, based on anticipated or experienced cognitive and emotional responses toward the intervention [16]. High community acceptance has been reported for ITNs [1]. However, there is growing evidence of inadequate coverage and that there is need to understand the barriers associated with use of bed nets [11, 17–20]. Community participation can help overcome these barriers by providing a better understanding of perceptions and behaviours [18, 21]. Community participation, however, is not just limited to providing insights. Community participation can range from noncompliance to complete ownership of an intervention [19]. Although appreciated, historically, community participation has not been fully utilized in malaria control, owing partly to the fact that community participation does come with its challenges [19]. However, a top-down approach, which is a characteristic of many malaria control programs, can lead to the failure of interventions, irrespective of the soundness of the intervention [12, 15, 22]. To be accepted and practiced, an intervention needs to be locally appropriate. This is only possible when it is developed through participatory approaches with all relevant stakeholders. The local community as the end user of an intervention will need to be given high priority for coproduction of intervention tools and strategies. Learning from the community, developing intervention with the community, and addressing people’s wishes and concerns can lead to better acceptability [15]. This in turn also provides access to local knowledge, expertise and labour for implementing the intervention [23–25].

Strategies that involve mosquito release have been of particular concern to communities [15]. Concerns range from fear of an increase in mosquito bites to failure of refractory mechanisms in the wild, disruption of food chain, evolution of more virulent pathogens, to possible transmission of new diseases [26]. A few examples of successful releases of genetically modified (GM) mosquitoes are now available but have been made possible only by acknowledging the need of community engagement [27, 28]. There is still significant concern regarding the release of GM mosquitoes in Asia, America and Africa which indicates that intensive engagement with communities and stakeholders and appropriately tailored communication strategies are required [29–33]. Strategies based on natural microbes that inhibit *Plasmodium* transmission in mosquitoes are expected to be perceived as relatively safe compared to those based on genetic modification. However, despite the different backgrounds of the strategies, communities may have the same concerns, which need to be addressed with community engagement. Community outreach activities resulted in increased acceptance of the release of mosquitoes infected with a bacteria *Wolbachia* for dengue control in Australia, Indonesia, Brazil and Malaysia [34–37].

*Microsporidia MB*, a recently discovered microsporidian is naturally found in *Anopheles* mosquitoes. It inhibits the mosquito from transmitting malaria parasite and is a promising candidate for transmission blocking [38]. Unlike *Wolbachia*, *Microsporidia MB* is stable in *Anopheles* and spreads in the mosquito populations by vertical (from mother to offspring) and horizontal (by mating) transmission [38, 39]. Other modes of horizontal transmission have not been identified but cannot be ruled out completely [40]. Extensive ongoing research will inform the development of the final *Microsporidia MB*-based transmission-blocking strategies, however, three scenarios can be anticipated: (i) infective *Microsporidia MB* spores released in *Anopheles* mosquito breeding sites, (ii) ovipositing or sugar feeding mosquitoes attracted by semiochemicals and infected with *Microsporidia MB*, and (iii) *Microsporidia MB*-infected males and females or only male mosquitoes released in the environment [40].

The *Microsporidia* MB-based transmission blocking strategy is at an early stage which is timely for developing a comprehensive stakeholder engagement process that results in the co-development of intervention packages and appropriate communication strategies. The aim of this rapid assessment was to obtain initial insights into sociodemographic factors, public health concerns, malaria awareness, management, and prevention practices that are associated with the willingness to accept and participate in a *Microsporidia MB*-based transmission blocking strategy that involves mosquito-release. The term “willingness to accept” was used to avoid confusion with “acceptance” as the strategy is still under development. Also, “willingness to participate in the implementation of the strategy” was to estimate expected active community participation [19].

## Methods

### Study site

**Fig. 1:**
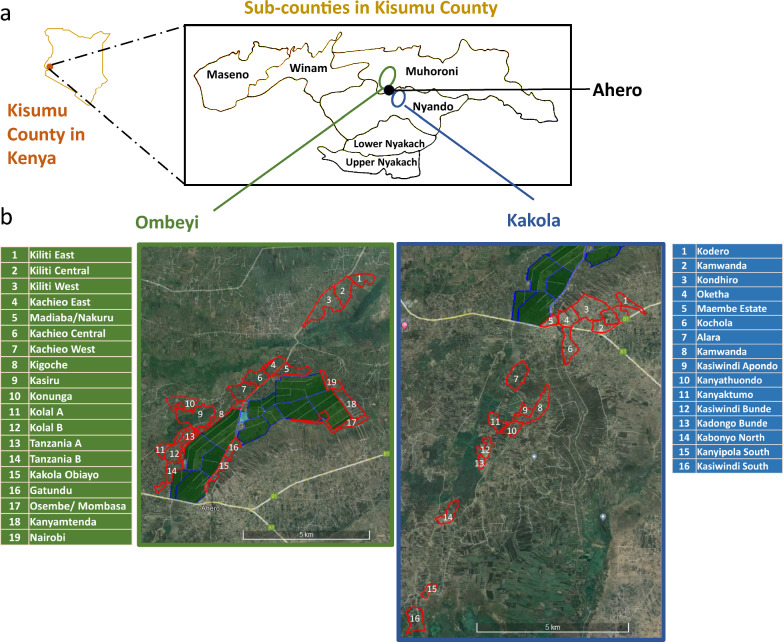
**a** Ombeyi and Kakola location in reference to the Sub-counties in Kisumu County, western Kenya **b** List and map of 19 Villages in Ombeyi and 16 villages in Kakola. Ahero irrigation scheme in blue for reference

The surveys were conducted in 35 villages in two administrative locations: Ombeyi and Kakola, near Ahero (0° 10ʹ N, 34° 54ʺ E), a town located in Kisumu County, western Kenya (Fig. 1a). Ahero is located along the Kisumu-Nairobi highway and serves as a town center for several villages surrounding the Ahero irrigation scheme (Ombeyi location, Sub-County Muhoroni, population 154,501) and South West Kano irrigation scheme (Kakola location, Sub-County Nyando, population 161,501) [41, 42]. The region experiences a modified equatorial climate characterized by long rains (March to May) and short rains (September to November). Agriculture results in employment for 70% of the population. Rice and several crops are grown year-round under irrigation using water from the Nyando, Nyatini and Ngadi rivers. The status of education is far below the national standards [43]. Malaria is the most prevalent ailment experienced by residents with an estimated 29–35 bites per person per night [44–47].

This study is part of the Symbiovector project based at the International Center of Insect Physiology and Ecology (*icipe*), Kenya that aims to develop a *Microsporidia MB*-based transmission blocking strategy. The project involves intensive indoor mosquito collections from the study area to assess the natural infection of malaria-transmitting mosquitoes with the microbe. The project team has been carrying out mosquito collections frequently in the Ombeyi location and, therefore, the administration and some households were aware of the project. However, the administration of Kakola location were not aware of the project and the stakeholder meetings, described below, was their first contact with the project team. The survey was done together with household mapping aimed at understanding the relationship between the environment and *Microsporidia MB* prevalence making it feasible to include a higher number of households.

### Study design

The study was quantitative and based on questionnaire administered at household level. The questionnaire consisted of structured, semi-structured and open questions (Additional file 1). The survey was carried out from June to November 2022.

### Stakeholder meetings and partnerships

Informal meetings were purposefully held to build trust and partnership with stakeholders to conduct the assessment. At least two meetings were held with the National Irrigation Authority (NIA), the Ministry of Health (MoH) and local administrations including the chief, assistant-chief and village elders. A community was defined as people living in the same geographical location that is also an administration unit, therefore, this study involved two communities: Ombeyi and Kakola [24]. Village level administrative maps were unavailable. In consultation with NIA and administration, a village list was compiled, and village boundaries were established by recording tracks of GPS coordinates around each village with the aid of village elders. In total 19 villages represented the Ombeyi community, and 16 villages represented the Kakola community (Fig. 1b). All households found in the selected villages were eligible to participate in the study. To ensure that communication for the upcoming household survey reached the household level, the research team presented the proposed survey in community meetings. The community meetings involved at least 20 household heads (HHs) per village who were nominated by the village elder on the basis of uniformity in village household coverage and their capacity to sensitize other villagers. These meetings were attended by a total of 321 male and 484 female participants. With a few exceptions, all the stakeholder meetings were in person, informal and conducted at designated offices, local vocational institutes, church compounds, rice drying yards or community evacuation centres.

Sub-county community health focal persons (SCHFP), community health assistants (CHAs) and community health volunteers (CHVs) assigned to the selected villages, by Ministry of health, were recruited as enumerators to conduct the survey. The recruited group consisted of 1 female and 1 male SCHFP, 13 female and 3 male CHAs, and 39 female and 5 male CHVs.

### Assessment tool

The data were collected and managed using REDCap (Research Electronic Data Capture), a platform designed to support data capture for research studies [48]. The data collection tool was a questionnaire administered at the household level to the household head or his/her designate aged > 18 years in the presence of the household head. The questionnaire was divided into four parts to collect information on (1) sociodemographic factors and housing (2) public health concerns (3) malaria awareness, management, and prevention practices and (4) willingness to accept and participate in a mosquito release strategy.

### Training, pretest and assessment exercise

Two-day training on ethical conduct and the use of the REDCap tool was organized for the enumerators: CHVs, CHAs and the SCHFPs of Sub-County Muhoroni and Nyando.The questionnaire was in English and, during the training, the background of each question was discussed with the enumerators so that they could explain the question to the households heads. There was no restriction on the use of Dholuo for administering the questions. Prior to the data collection exercise, the tool was pretested by each CHV visiting two homesteads to administer the questionnaire and submit the collected data for approval to the CHAs followed by verification by the study team. The CHVs were encouraged to share their challenges after the pretest and the tool was modified accordingly. Four of the CHVs were unable to confidently operate the handheld devices and were accommodated with a printed version of the questionnaire for manual data collection. Each CHV aimed to visit 10 households/day to administer the questionnaire to household heads or their designates. A WhatsApp group was created for timely response to issues or questions arising within the team.

### Ethical considerations

The household heads (HHs) were interviewed after providing consent. The study was approved by the scientific ethics review unit (SERU) of Kenya Medical Research Institute (Non-KEMRI protocol number 4520).

### Data analysis

Qualitative data were analysed in NVivo v.12 using thematic analysis [49]. The data were read for familiarizing, after which a codebook was developed through deductive use of topic guides and inductive open coding of a sample of responses. A second tier of axial coding was carried out through close reading the underlying data in each code and merging of redundant codes and, last, clustering linked codes to broader and coherent categories. Text for the manuscript was then developed by identifying key themes from the broad categories and building linkages between them. Compelling verbatim accounts of verbal utterances were captured. Sections of qualitative responses were subjected to word queries to identify recurrent themes and facilitate the construction of word clouds.

Quantitative data were analysed descriptively using IBM Statistics SPSS Software (IBM Corp. IBM SPSS Statistics for Windows, Version 27.0. Armonk, NY: IBM Corp.). For associations between sociodemographic factors, public health concerns, malaria awareness, management, and prevention practices, odds ratios (OR) and 95% confidence intervals (95% CI) were calculated using Mantel–Haenszel statistics [50].

Willingness to accept and participate was evaluated based on the response “yes”. To determine factors that are related to willingness to accept and participate in a mosquito release strategy, odds ratios with 95% confidence interval (CI) were calculated first in a bivariate analysis. Then, only the significant (p < 0.05) variables were included in a multivariate logistic regression model with stepwise Wald backwards method to generate adjusted odds ratios (AOR) with 95% confidence intervals (95% CI).

## Results

### Sociodemographic factors

The data were collected from a total of 8108 households in the Kakola (47.5%, n = 3851) and Ombeyi (52.5%, n = 4257) communities (Table 1). The total population living in these households was 37,860 people. None of the household heads (HHs) refused to participate in the assessment. Majority of the HHs were male (67%, n = 5432), within the age bracket of 30–49 years (48%, n = 3892) and married (81%, n = 6567). In general, 66% (n = 5351) of HHs were educated at or above primary level, with HHs in Ombeyi being less likely [OR (95% CI): 0.53 (0.48–0.58)] to be educated at or above primary level compared to Kakola. The main occupation was farming, with more farmers living in Ombeyi compared to Kakola [OR (95% CI): 1.6 (1.5–1.8)].

**Table 1:** Sociodemographic characteristics of household heads (HHs) interviewed

Factors	n (%)
Total respondents	8108
Location/community	
Kakola	3851 (47%)
Ombeyi	4257 (52%)
Sex	
Male	5432 (67%)
Female	2676 (33%)
Age bracket (years)	
18–29	973 (12%)
30–49	3892 (48%)
50–64	1703 (21%)
65 +	1541 (19%)
Marital status	
Monogamous	5838 (72%)
Polygamous	730 (9%)
Widowed	973 (12%)
Single	568 (7%)
Education level	
No formal education	770 (9%)
Incomplete primary	2027 (25%)
Primary	2919 (36%)
Secondary	2027 (25%)
Tertiary	405 (5%)
Occupation	
No job	8 (0.1%)
Formally employed	649 (8%)
Casual laborer	2189 (27%)
Farmer	3770 (46%)
Self-employed/small business	1378 (17%)

**Fig. 2:**
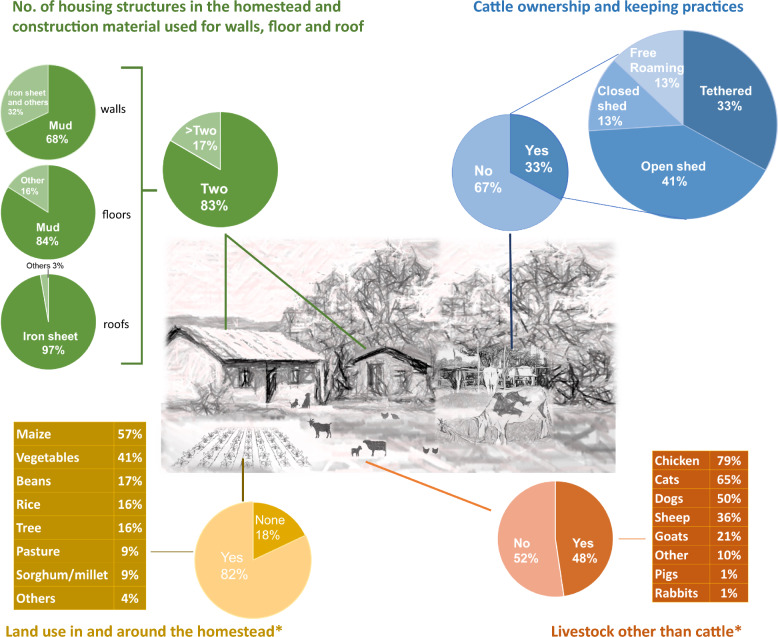
A typical homestead in the study site and the housing structure, cattle and livestock ownership and land use characteristics. *Responses after “yes” were not exclusive/Multiple selections were possible

A typical homestead (Fig. 2) consisted of two housing structures (83%, n = 6730), both with mud walls (68%, n = 4576) and floor (84%, n = 5653) and iron sheet roof (97%, n = 6528). Only 33% (n = 2676) of the households kept cattle. On average, cattle owners had 4 animals (range 1–150) that were kept in the homestead either tethered in the open (33%, n = 883) or in a roof-less shed (41%, n = 1097) at night. Livestock, other than cattle, were kept in 48% (n = 3892) of the households with chicken, sheep, or goats of economic significance. Livestock ownership was 30% higher in Ombeyi (n = 1565) compared to Kakola (n = 1087) [OR (95% CI): 1.3 (1.2–1.45)]. The land inside and around the homestead was primarily used for subsistence farming of multiple crops.

### Public health concerns

Malaria was considered a severe public health concern by most respondents in both locations (Table 2A). In Ombeyi location, pneumonia and typhoid were also reported as important health problems, and some of the respondents felt that the next hospital was far and the medicine supply was inadequate. Thematic analysis showed that in Kakola, poverty emerged as an important challenge for public health.

**Table 2:** Malaria awareness and prevention practices

Factors	n (%)
A. Malaria a public health issue	
Perceive malaria to be a severe public health issue	5529 (68%)
Perceive malaria to be a moderate public health issue	2412 (30%)
Perceive malaria to be a low public health issue	121(1.5%)
Perceive malaria to be not a concern	34(0.4%)
B. Know mosquitoes transmit malaria	
Only mosquito bites transmit malaria	6262 (77%)
Mosquito bites and/or other factors transmit malaria	1833 (23%)
C. Known malaria prevention methods*	
Bed nets	7712 (95%)
Mosquito coils	4769 (59%)
Indoor spraying	2935 (36%)
Medications	2930 (36%)
Repellents	2495 (31%)
No control method can prevent malaria	86 (1%)
Symbiont/microorganism-based methods	94 (1%)
Other	89 (1%)
None of these	43 (0.5%)
D. Malaria prevention method used in the last 30 days*	
Bed nets	7704 (95%)
Clearing bushes around the house	2848 (35%)
Mosquito coils	2653 (33%)
Draining stagnant water	2449 (30%)
Medication	1936 (24%)
Repellents	1356 (17%)
Insecticide spray	874 (11%)
None	29 (0.4%)
Other	20 (0.2%)
E. Mosquito net use	
Daily	8056 (99.4%)
Every household member uses a bed net	7937 (98.2%)

*Answers were not exclusive/multiple choices were possible

### Malaria burden, awareness, management, and prevention practices

In total 13,544 cases, diagnosed at a health facility within the last six months, were reported. Among them 37% (n = 5091) were cases of multiple infections in the same person.

The awareness that mosquito bites transmit malaria was high (77%, n = 6262), but 23% (n = 1833) of the respondents also believed that in addition to mosquito bites, other factors such as contaminated water, and cold or rainy weather contributed to malaria transmission (Table 2B).

The majority (86%, n = 7011) of the respondents perceived that children under the age of 5 years and pregnant women were at a higher risk of malaria. Few respondents (17%, n = 1351) believed that elderly people or, in general, everyone is at risk of malaria. The prevalence of malaria in the region was attributed to stagnant water resulting from rain and rice cultivation in both the Kakola and Ombeyi locations.

*“Stagnant water occurred during rainy season and rice farming…..” Kakola-Female-72 years*

### Treatment-seeking practices

The most common malaria drug used was AL (Artemether-lumefantrine, Coartem) tablets (78%, n = 3003). Hospital visits/consultations involving CHVs were also reported by Kakola respondents (4%, n = 135) but not as often by respondents in Ombeyi (51%, n = 2168). In both locations, some of the respondents also indicated self-medicating with common malarial tablets and pain killers before visiting a hospital (4%, n = 302). Debilitation due to illness was the main driver for seeking medical intervention at a health facility while limited financing was the main barrier.

The medicines for malaria treatment received at health facilities by respondents were Coartem, Paracetamol, ACT (artemisinin-based combination therapy), anti-malarial, injection, and Panadol as well as Fansidar (sulfadoxine and pyrimethamine) tablets. In Kakola, the majority indicated having received ACT tablets for treatment (4%, n = 147), while in Ombeyi most participants reported having been provided with Coartem (5%, n = 218) for sick children, aged less than 5 years.

Very few respondents indicated that they would resort to herbal/natural alternatives (0.1%, n = 10), or their religious beliefs (n = 3) in addition to medication. Women were reported as the main decision-makers (50%, n = 4049) of whether a family member with malaria-like symptoms sought treatment. Men were reported as the decision-maker half the time as women (24%, n = 1724). In the two locations, both female and male respondents perceived it was mostly the mother who made the decision. Fewer treatment-making decisions were made jointly by both parents ((12%, n = 972) or grandmother in the family (14%, n = 1131).

### Prevention practices

The use of bed net (95%, n = 7704) was the most common method for preventing malaria, followed using mosquito coils (59%, n = 4769) (Table 2C, D). A high daily use (99%, n = 8063) of bed nets by every family member was recorded (Table 2E). Other prevention methods used included clearing bushes, draining stagnant water and smoking cow dung. Very few respondents (< 1%, n = 20) reported the need to ensure warm clothing, clean hands, bathing with hot water and burn plastic bags to prevent malaria. Only 94 (1%) HHs were aware of a microbe-based malaria control method.

Nearly three quarters of the respondents (72%, n = 5829) reported spending more than USD7 annually on malaria prevention. No government-led malaria control initiatives at household level were reported for the last six months. However, when asked specifically if they had been given free treatment, issued a free bed net, or their houses sprayed by government, 7% (n = 587) of the participants reported spraying, 13% (n = 1022) reported issuing of free bed nets at home and 8.5% (n = 692) reported receiving a bed net from a health facility as an expectant mother.

### Willingness to accept and participate in implementing a mosquito-release strategy

**Fig. 3:**
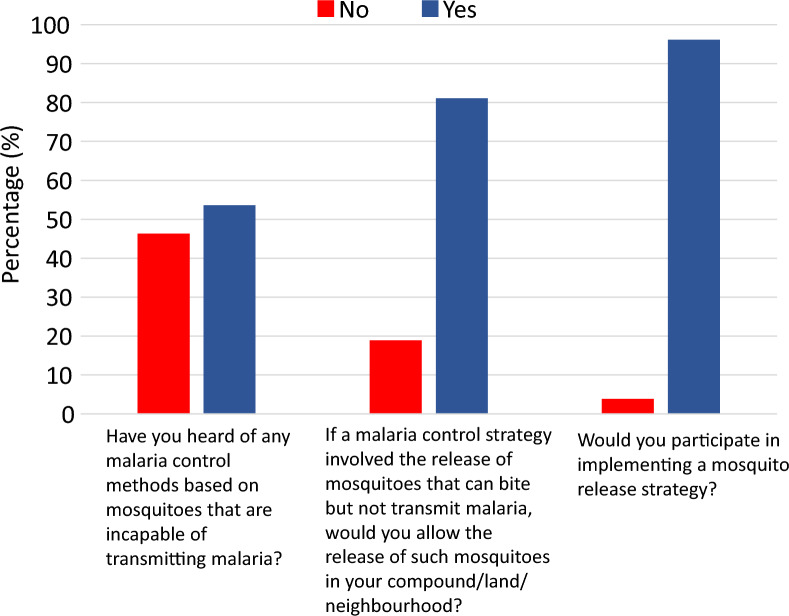
Response of household heads regarding awareness to transmission-blocking malaria control methods, acceptance to mosquito releases and willingness to participate

Half of the HHs (54%, n = 4349) were aware of malaria control methods that involve mosquitoes that are incapable of transmitting malaria (Fig. 3). When asked if they would allow the release of mosquitoes that can bite but are naturally unable to transmit malaria in their compound, land or neighborhood, 81% (n = 6565) responded “yes”. In addition, a greater percentage of HHs (96%, n = 7738) reported willingness to participate in a mosquito release strategy.

The hesitation to allow the release of mosquitoes in their surroundings was due to expressed fear and discomfort indicating that they did not trust that the released mosquitoes would not transmit malaria. The mosquito release was assumed to increase the number of mosquitoes which in turn would increase the nuisance and bites. Few participants speculated that the “new mosquitoes” may cause skin diseases and introduce new diseases in addition to malaria, putting children and elderly people at an increased risk.

**Fig. 4:**
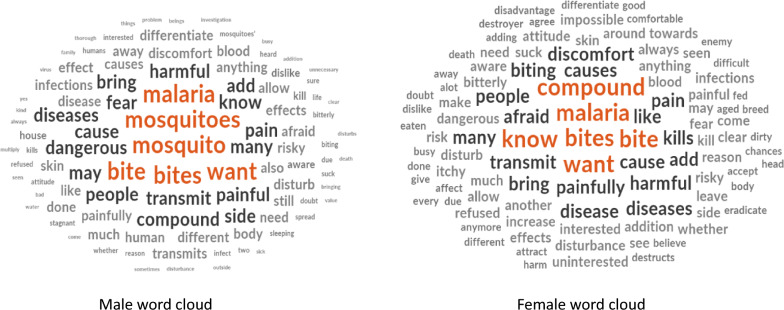
Word clouds of male and female household heads indicating the recurring perceptions for not accepting mosquito releases

The word clouds indicate that the recurring perceptions of females and males were similar and were mainly the fear of more and painful mosquito bites. However, women appear more concerned about the release of mosquito in their “compound” or in close proximity to their home and family (Fig. 4).

### Factors associated with the willingness to accept and participate in implementing a mosquito release strategy

Bivariate analysis showed that willingness to accept a mosquito release strategy by HHs was similar for Ombeyi and Kakola (OR 1.1, 95% CI 1.01–1.3) but those of Ombeyi were more willing to participate (OR 19, 95% CI 13–31) than were those of Kakola (Table 3). Compared to men, women were less willing to accept (OR 0.7, 95% CI 0.6–0.8) or participate (OR 0.7, 95% CI 0.6–0.9). Age had no impact on the willingness to accept or participate. HHs with a primary level or higher education were less likely to accept (OR 0.7 95% CI 0.6–0.8) and to participate (OR 0.5 95% CI 0.4–0.7) in mosquito release strategy compared to those with no or some primary education.

**Table 3:** Bivariate analysis of factors associated with willingness to accept and participate in implementing a mosquito release strategy

	Willingness to accept a mosquito release strategy			Willingness to participate in implementing a mosquito release strategy		
	Yes n (%)	Total n	0R* (95% CI)	Yes n (%)	Total n	0R* (95% CI)
Location						
Kakola^a^	3084 (80)	3846		3553 (92)	3844	
Ombeyi	3492 (82)	4249	**1.1 (1.01–1.3)**	4222 (99.5)	4240	**19 (12–31)**
Sex						
Male^a^	4477(83)	5396		5204 (96.5)	5388	
Female	2111(78)	2712	**0.7 (0.6–0.8)**	2584 (95)	2709	**0.7 (0.6–0.9)**
Age (Years)						
18–29^a^	814 (82)	995		956 (96)	994	
30–49	3141 (81)	3861	0.97 (0.8–1.2)	3698 (96)	3856	0.9 (0.6–1.3)
50–64	1365 (82)	1669	0.99 (0.8–1.2)	1605 (96)	1666	1.04 (0.7–1.6)
65 +	1228 (80)	1528	0.91 (0.7–1.1)	1477 (97)	1527	1.2 (0.8–1.8)
Education level						
No formal education	583 (76)	771	**0.6 (0.5–0.9)**	746 (97)	769	**2.4 (1.4–4.3)**
Incomplete primary	1705 (85)	1997	1.2 (0.9–1.6)	1952 (98)	1997	**3.3 (2.0–5.4)**
Primary	2320 (80)	2883	0.9 (0.7–1.1)	2754 (96)	2877	**1.7 (1.1–2.6)**
Secondary	1643 (80)	2047	0.9 (0.6–1.1)	1956 (96)	2045	**1.7 (1.09–2.6)**
Tertiary^a^	337 (82)	409		379 (93)	408	
Occupation						
No Job	1 (10)	10	**0.36 (0.004–0.3)**	10 (100)	10	**–**
Employment	574 (85)	676	**1.8 (1.4–2.3)**	647 (96)	674	**2.1 (1.3–3.3)**
Casual laborer	1862 (84)	2211	**1.7 (1.4–2.0)**	2123(96)	2210	**2.7 (1.6–2.9)**
Farmer	3067 (81)	3787	**1.4 (1.2–1.6)**	3699 (98)	3782	**4 (3.0–5.3)**
Self-employed/small business^a^	1034 (76)	1365		1251 (92)	1362	
Perception of malaria as a public health issue						
Severe^a^	4578 (83)	5529		5478 (99)	5527	
Moderate	1877 (78)	2412	**0.7 (0.6–0.8)**	2157 (89)	2412	**0.08 (0.5–0.1)**
Low	96 (79)	121	0.8 (0.5–1.2)	117 (97)	121	**0.26 (0.9–0.7)**
Not a concern	28 (82)	34	0.9 (0.4–2.3)	33 (97)	34	0.29 (0.04–2.2)
No. of malaria cases in the family over the last six months						
0^a^	579 (79)	735		716 (98)	734	
1–3	4772 (80)	5939	1.1 (0.9–1.3)	5669 (95)	5937	**0.5 (0.3–0.9)**
> 3	1152 (87)	1325	**1.8 (1.4–2.3)**	1301 (98)	1324	1.4 (0.8–2.6)
Knowledge on what transmits malaria transmission						
Mosquito bites and/or other factors^a^	1420 (77)	1843		1780(97)	1837	
Mosquito bites only	5168 (82)	6265	**1.4 (1.2–1.6)**	6008(96)	6260	0.8 (0.6–1.01)
Awareness of control methods based on mosquitoes that cannot transmit malaria						
No^a^	3035 (81)	3758		3517(94)	3749	
Yes	3553 (82)	4349	1.06(0.95–1.2)	4271(98)	4348	**3.6 (2.8–4.7)**

^a^Reference group

*Odds ratio (bold values indicate a significant difference, p < 0.05)

Occupation did not influence acceptance, but HHs related to agriculture were more willing (OR 2.6 95% CI 2–3.4) to participate then were those employed or running a business. HHs that considered malaria a moderate, low or not a public health issue were also less likely to accept (OR 0.7 95% CI 0.6–0.8) or participate (OR 0.07 95% CI 0.06–0.1) for a mosquito release strategy. The number of malaria cases reported were categorised for analysis (1 = no case; 2 = 1–3 cases; 3 ≥ 3 cases). Higher number of malaria cases experienced in the household over the last six months increased the willingness to accept (OR 1.3 95% CI 1.1–1.5) and participate (OR 1.65 95% CI 1.3–2.1) in a mosquito release strategy. Respondents who knew that only mosquito bites transmit malaria, and not factors such as contaminated water or cold weather, were also more likely to accept (OR 1.4 95% CI 1.2–1.6) but less likely to participate (OR 0.7 95% CI 0.6–1.01) in a mosquito release strategy. Participants who were aware of mosquito control methods involving mosquitoes which are unable to transmit malaria were no different from unaware ones in accepting but were more willing to participate (OR 3.7, 95% CI 2.8–4.7) in a mosquito release. Due to the high level of bed net awareness and use, these factors could not be analysed.

After adjusting for other factors with multivariable analysis (Table 4) sex, education occupation, perception of malaria severity as public health issue, experience with more malaria cases and knowledge that malaria is transmitted by mosquito bites were found to be associated with the willingness to accept. Location, occupation, perception of malaria severity as public health issue, experiencing higher malaria cases, knowledge that malaria is transmitted by mosquito bites and awareness of control methods that involve mosquitoes which are unable to transmit malaria were associated with the willingness to participate in the intervention.

**Table 4:** Factors associated with willingness to accept and participate in implementing a mosquito release strategy after multivariate analysis

Factors	Willingness to accept a mosquito release strategy		Willingness to participate in the implementation of a mosquito release strategy	
	AOR* (95% CI)	p-value	AOR* (95% CI)	p-value
Resident of Ombeyi location			22 (13–36)	< 0.001
Being of female sex	0.8 (0.7–0.9)	< 0.001		
Incomplete primary education level	1.6 (1.2–2.2)	0.002		
Occupation				
Employment	1.8 (1.4–2.4)	< 0.001		
Casual labourer	1.6 (1.4–2.0)	< 0.001	1.4 (1.02–2.0)	0.040
Farmer	1.3 (1.1–1.5)	< 0.001	2.0 (1.4–2.7)	< 0.01
Perceiving malaria as a moderate public health issue	0.7 (0.6–0.8)	< 0.001	0.07(0.05–0.1)	< 0.001
Perceiving malaria as a low public health issue			0.07(0.02–0.3)	< 0.001
1–3 malaria cases in the family over the last six months			0.15 (0.09–0.2)	< 0.001
> 3 malaria cases in the family over the last six months	1.7 (1.3–2.2)	< 0.001	0.2 (0.1–0.4)	< 0.001
Knowledge that only mosquito bites transmit malaria transmission	1.4 (1.2–1.6)	< 0.001	0.7 (0.5–0.9)	0.040
Aware of control methods based on mosquitoes that cannot transmit malaria			5.2 (3.9–7.0)	< 0.001

*Adjusted odds ratio

## Discussion

While there was low awareness in the community of transmission-blocking strategies involving the release of non-malaria transmitting mosquitoes, the majority of the interviewed respondents indicated that they were willing to accept such a strategy. While encouraging, it is important to understand the factors contributing to the unwillingness of the participants. The approximately 20% of respondents who were hesitant of the release of non-malaria transmitting mosquitoes, while not a majority, can have a significant impact on whether or not a strategy is accepted and adhered to, particularly if they represent influential community members. It is notable that the awareness for strategies that involve release of mosquitoes in the study site was considerably higher compared to similar studies elsewhere. Only 5% awareness was reported for *Wolbachia*-based strategy in Puerto Rico [14] and 6% for modified mosquitoes-based technology in Tanzania [30]. It is, however, comparable to the acceptance level (96%) for mosquito release in Burkina Faso, but this was following intensive knowledge building in the community prior to release of non-genetically modified mosquitoes [51]. The acceptance for mosquito release in Florida, US was reported to be 57% when only households that were aware of sterile male mosquito release were considered [52]. Altogether, these findings highlight the need for intensive community engagement to increase awareness of the community on strategies that involve mosquito releases in the field.

Perception of severity of malaria is associated with knowledge and experience with malaria [53, 54]. Treatment seeking behavior is influenced by residence (rural or urban), caregivers age, knowledge of malaria, perceived malaria risk, and perceived barrier to seek treatment [55, 56]. This study shows that the perception of the severity of malaria and treatment-seeking behaviour can vary even in neigbouring communities that have the same epidemiology of the disease. This study showed that Ombeyi and Kakola, despite their furthest villages being only 15 km away from each other, differed in several aspects. Compared to those from Kakola, participants from the Ombeyi community were less educated, more dependent on farming and had higher cattle and livestock ownership. Concerning treatment seeking, Ombeyi residents were more likely to go to the health facility compared to Kakola. In Kakola, economic inability to meet needs appeared to be the main reason for delaying the treatment. Treatment in Ombeyi, maybe affordable due to the availability of livestock that can be sold during emergencies. Ombeyi HHs were also more willing to participate in a mosquito release strategy. This difference in the responses of the two communities indicates the importance of formative research at community level to ensure that the communication material is adapted to their unique aspects.

Over 50% of the respondents reported that it is exclusively mothers that make the decision for treatment seeking. Only fewer respondents identified joint decision making by both parents (12%). This is higher than the 33% female household heads that participated in the survey and indicates a general appreciation that women are the decision makers, which can be entirely their own decision or a joint decision. The present findings agree with results of other authors which identified mothers as primary decision makers for health care-seeking as they are believed to have a maternal instinct especially for the vulnerable younger children than others and are often considered to be educated on the healthcare during hospital visits [57]. Although, the malaria prevention and treatment decisions in the household were associated with the female caregiver, women were less willing to accept the mosquito release.

The interviews were targeted at household heads of which only one third of these participants were women, this could have led to the underrepresentation of women’s opinions. However, women living in households with a male head may not be involved in decision making for mosquito releases. This can be explained by observations in many communities where men are known to dominate decision-making especially in less routine activities or engagements. This domination of males in decision-making at the household level is often observed in households with partners with varied preferences and bargaining power [58]. This study also showed that the reason for the low acceptance and willingness of the women to participate was the fear of higher number of mosquitoes in close proximity to their family and, therefore, leading to more mosquito bites on family members. Low acceptance of GM mosquito releases was also reported in women, although, the underlying factors were not identified [52, 59]. The role of women at the household, community and programmatic levels in vector control is increasingly being recognized [25]. Women are equally effective at implementing vector control as men. In addition, they have access to more information at the household levels and are meticulous (identifying breeding sites, less breakage or downtime in monitoring tools); moreover, as their sense of ownership increases, vector control becomes more sustainable [60, 61]. Therefore, it is important to understand the factors that influence women’s opinions on vector control interventions and how to address them [62]. One recognized factor is interference in the domestic domain, such as the entrance of public health representatives or changes made within the house and compound, e.g., eliminating breeding containers, screening houses or plastering walls [62]. This highlights the need to mindfully involve women in community engagements and to develop communication materials that addresses their concerns to promote women’s acceptance and participation in mosquito releases [63].

It is expected that higher perceived risk would relate to greater willingness to accept and participate in vector control intervention. This was evident from the greater acceptance and willingness to participate in mosquito release of HHs which also perceived malaria as a severe public health issue. Practicing disease prevention can also be used as an indicator of higher risk perception. In a previous study, the use of repellents was found to be associated with increased support for a *Wolbachia*-based population suppression strategy [14]. However, a lack of correlation has, also, been found between risk perception and acceptance and support for vector control interventions [64]. This study recorded a very high level of bed net use; therefore, it was not possible to determine whether bed net use has any association with attitude towards a mosquito release strategy. Despite a high and regular use (> 98%) of bed nets, 37% of the total population was reported to have had at least one malaria infection in the previous six months. However, this is not surprising as similar studies have reported high malaria infection rates in communities characterized by high use of bed nets. It is possible that bed net use is high but suboptimal due to improper tucking or bed net attrition over time [17]. The study area, also, has a high population of *Anopheles arabiensis*, which are exophilic, thus, bed nets might not provide the desired protection from bites by this mosquito species [44].

Experience with more than three malaria cases in the family within the last six months also increased the acceptance and willingness to participate in a mosquito release strategy. A similar result was shown in Guatemala where experience with dengue increased the acceptance of integrated control for dengue including larval source management, treated curtains and drum covers, tools that rely heavily on community participation to be effective [65]. Experience, as a factor associated with acceptance for mosquito releases, is particularly relevant in areas with seasonal malaria transmission where malaria cases are rare or absent over months at a time. Reduced bed net usage has been reported in such regions due to a perceived lack of mosquitoes [66]. This finding suggested that intensive community engagement may be required if mosquito release is timed at the beginning of the malaria transmission season in regions with seasonal malaria. This study was carried out in a region with a high mosquito population; therefore, formative work in regions with seasonal malaria is needed to understand the link between malaria epidemiology and willingness to accept it.

Participants with knowledge that malaria is transmitted by only mosquito bites and not factors such as cold weather or contaminated water were more likely to accept but less likely to participate in the implementation of transmission-blocking strategies. Similar factors have also been reported to cause malaria transmission elsewhere [67]. Interestingly, higher education level was associated with less willingness to accept. Although concerns about mosquito release have been raised by educated people, generally higher education is related to higher acceptance [13, 52, 68]. However, these results suggest the importance of understanding the difference in the concerns raised by community members with different education levels. The educated people in the community are influential and can be resourceful for implementing a strategy if their concerns are addressed appropriately.

Half of the respondents were aware of malaria control interventions that are based on mosquitoes that are unable to transmit malaria and the same respondents were also more willing to participate in the intervention. Only 1% of HHs indicated that they were aware of microbe-based transmission-blocking strategies. Although, in our community meetings with HHs we presented the *Microsporida MB* project verbally to almost 10% of the HHs who participated in the assessment. Although, one explanation could be that the nomination of HHs by some community elders was suboptimal, low awareness has been previously reported despite outreach activities [52]. This shows the need to utilize several outreach methods, such as posters and radio, in community sensitizations.

The high willingness to participate in the implementation of mosquito release strategy may have been influenced by job prospects [69]. New projects create job opportunities and contribute to tangible assets such as income and property and intangible assets such as skills, knowledge, social network and personal development [70]. This understanding, however, does bias the information given by the community. It is also important to reiterate here that two thirds of the respondents were male and naturally more inclined towards the job prospects than women who were concerned about health implications.

Some limitations of the study include the following (i) the Ombeyi community was not entirely naive as the study team carries out extensive mosquito collections in the locations and there is a certain level of trust on the institute that is developing the mosquito-release strategy, (ii) the HHs’ opinion may not reflect the opinion of the entire household and is biased toward males (iii) the study was conducted in a region with high mosquito densities which influences the acceptance of mosquito control intervention and (iv) the household heads showed higher willingness to accept and participate despite almost half being unaware of any technology that uses mosquitoes unable to transmit malaria. As mentioned above, this might be driven more by the project presence in community and expectations of benefits. Therefore, there is a need to further the study where the intervention is explained to the communities.

## Conclusion

Overall, the study revealed a high-level of willingness to accept and participate in a *Microsporidia MB*-based mosquito release strategy in a malaria endemic area. The willingness to accept and participate is influenced by several factors such as the community, disease risk perception, sex, education level, knowledge, and experience with malaria. Further research will need to focus on understanding the concerns of women, educated, and employed community members and the factors that contribute to lower disease risk perceptions. Also, this baseline level cannot be perceived as the actual acceptance, but a baseline from which to build community engagement and educational materials or strategies. Therefore, similar studies need to be carried out in areas that are naïve and with different malaria epidemiology. This will improve the understanding of *Microsporidia MB*-based mosquito release strategy from local perspectives and lead to the development of effective communication strategies.

### Supplementary Information

**Additional file 1.** RedCap Tool.

## Data Availability

The datasets analysed during the current study are available from the corresponding author on reasonable request.
